# Arbuscular mycorrhizal fungi as a hub for soil carbon transformation in intercropping systems: a review of microbial mechanisms and ecological significance

**DOI:** 10.3389/fmicb.2026.1929217

**Published:** 2026-08-28

**Authors:** Runzhi Zhang, Jingjing Yang, Yixian Liu, Lin Shi, Haiyan Yang, Runtong Li, Xinyu Zhang, Hongxue Wang, Guoling Ren

**Affiliations:** 1 School of Biotechnology, Daqing Normal University, Key Laboratory of Applied Chemistry and Technology in Oilfield, Daqing, China; 2 Hebei University, Baoding, China

**Keywords:** arbuscular mycorrhizal fungi, carbon transformation, intercropping, plant-microbe symbiosis, soil organic carbon

## Abstract

Arbuscular mycorrhizal fungi (AMF) play a pivotal role in soil organic carbon (SOC) dynamics by channeling plant-assimilated carbon into both labile and recalcitrant pools. In intercropping systems, AMF form symbiotic associations with host plants and facilitate nutrient exchange across the plant-fungus-soil continuum, which consequently enhances soil nutrient availability. Through transforming photosynthetic carbon into diverse organic fractions, AMF exert a dual influence on SOC reserves by promoting both carbon stabilization and decomposition. This review synthesizes current evidence for a conceptual framework centered on AMF life-history strategies, proposing that trade-offs between plant growth promotion and SOC storage are context dependent and modulated by fungal functional traits and community composition. Elucidating AMF mediated carbon transformation in intercropping systems is therefore critical for understanding carbon turnover mechanisms under diversified cropping regimes. Such knowledge not only supports yield improvement, soil structural reinforcement, and ecological restoration but also provides a theoretical foundation for developing sustainable soil management and rehabilitation strategies.

## 1 Introduction

Intercropping is a common practice in agricultural production that can improve nutrient use efficiency, enhance crop yield and quality, ameliorate soil structure, suppress weeds and pests, and increase the stability of agroecosystems through the synergy between plants and soil microorganisms. Intercropping not only boosts crop yield but also maximizes the use of aboveground light, groundwater, and soil nutrients (Rosa-Schleich et al., 2019; Tamburini et al., 2020). Extensive analytical and experimental data indicate that shifts toward diversified cropping systems, such as intercropping, have substantially altered soil organic matter and nutrient contents in agricultural fields (Techen et al., 2020).

Evidence indicates that, compared with monocropping, altered aboveground plant composition promotes macroaggregate formation and increases organic matter retention on clay mineral surfaces, leading to greater stabilization of soil carbon (Chen et al., 2017; Zhang et al., 2014; Liu et al., 2019). Meanwhile, intercropping affects both the quantity and chemical composition of litter and root exudates, which implies that newly input carbon may trigger either a positive or a negative priming effect on SOC. Through this priming, the mineralization and build-up of soil organic carbon are regulated, constituting an important avenue by which cropping system design influences overall SOC stocks. However, these studies have several limitations.

Soil organic carbon serves as a key indicator of soil quality and land productivity, and is closely linked to cropping patterns, tillage practices, and nutrient management. Some studies have shown that intercropping, by enhancing species diversity, promotes carbon sequestration and thus increases SOC content (Cong et al., 2015; Xiang et al., 2019). However, other studies have found that intercropping reduces SOC content, and that the organic carbon inputs entering the soil under intercropping systems decompose rapidly, which is unfavorable for SOC stabilization (Hu et al., 2021; Maia et al., 2019). Consequently, the effects of intercropping on SOC remain uncertain, highlighting the need for further in-depth investigation.

Some studies primarily focused on the responses of SOC content and related indicators to cropping patterns, tillage practices, and nutrient management (Luo et al., 2020; Li et al., 2021). However, the process level mechanisms underlying soil carbon transformation under intercropping systems have not been thoroughly investigated. As soil carbon transformation is subject to changes in vegetation, soil properties, and microbial communities, the characteristics of plants, soil, and microorganisms interact to influence the decomposition and stabilization of organic matter. Nevertheless, which factors dominate these processes under different cropping patterns remains unclear. Therefore, there is an urgent need to target the less-studied intercropping systems and to comprehensively investigate how changes in cropping practices affect soil carbon transformation processes and their underlying mechanisms.

**Figure 1:**
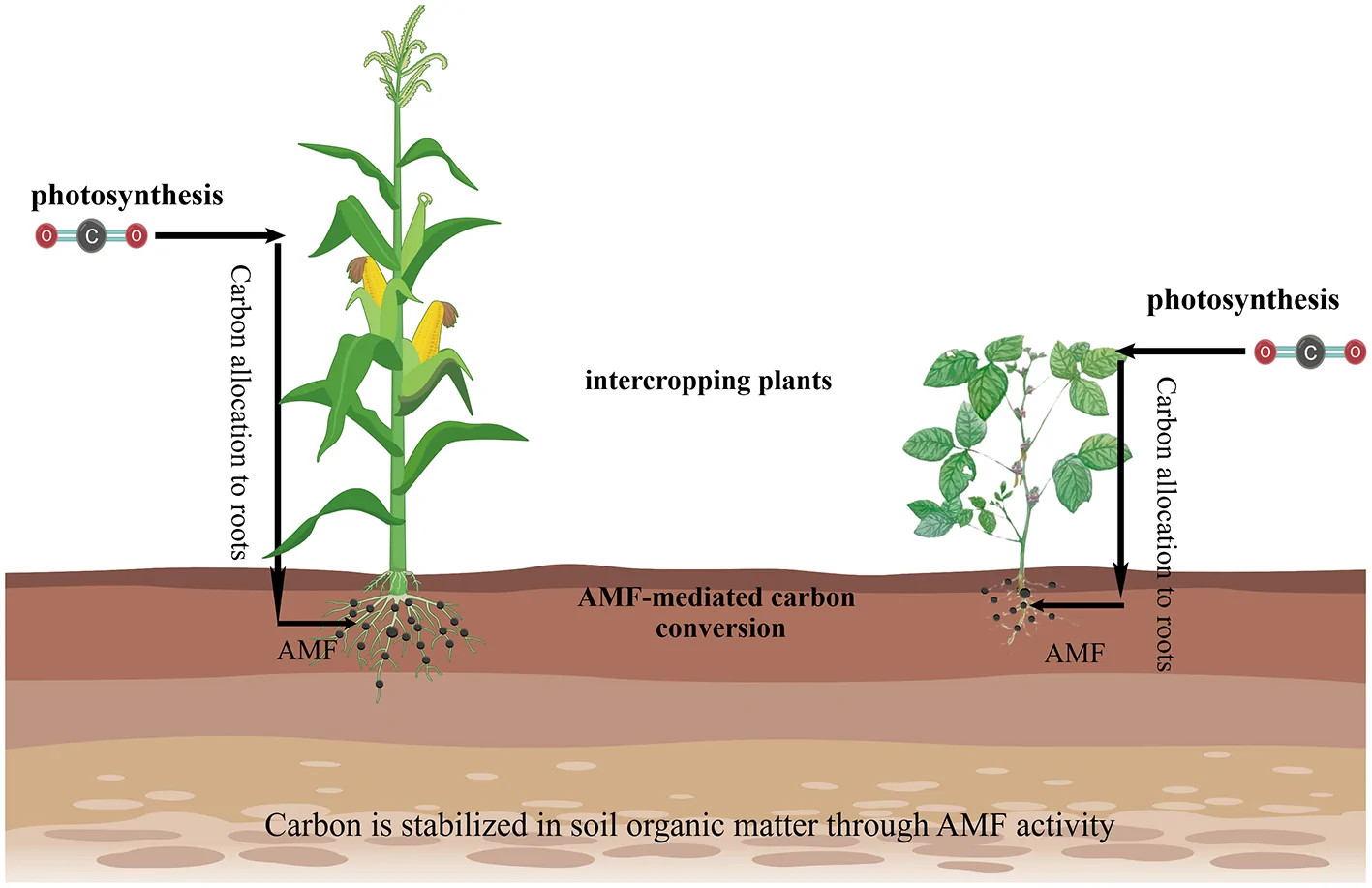
Schematic illustration of the symbiotic relationship between arbuscular mycorrhizal fungi (AMF) and plant roots.

Arbuscular mycorrhizal fungi, as symbiotic partners of plants, exert an influence on rhizosphere microecology and regulate the dynamics of the microbial community in the rhizosphere soil through their reciprocal interactions with host plants (Figure 1). Firstly, these fungi are capable of modifying both the host root system morphology and the rhizosphere microbial assemblage. They effect such modifications by extending root length, augmenting stem diameter, and promoting lateral root formation, as well as by facilitating water and nutrient capture and strengthening the host's ability to withstand adverse conditions (Sugiura et al., 2020; Rillig et al., 2020). Secondly, AMF are capable of stabilizing soil structure and altering soil physicochemical properties. They effect these changes by exuding organic acids or liberating protons in the rhizosphere, which consequently elevates soil organic carbon content, ameliorates soil quality, and expands the taxonomic and functional diversity of the rhizosphere microbial community (Wu et al., 2020; Kapoulas et al., 2019).

Therefore, this minireview advances four interconnected hypotheses that together form a conceptual framework for understanding AMF-mediated soil carbon transformation under intercropping systems: (1) Trade-offs between AMF induced plant growth gains and SOC storage are context-dependent, modulated by soil fertility, AMF species identity, and environmental conditions, such that enhanced nutrient acquisition by host plants may come at the cost of reduced carbon persistence in certain scenarios but not universally. (2) AMF tend to compensate for these trade-offs by depositing newly fixed photosynthetic carbon into the soil pool, but the extent of this compensation varies considerably depending on the life-history strategy of the AMF species involved. (3) Strong mutualist AMF that promote plant growth primarily through mobilization of mineral-associated organic matter exert a more negative effect on soil carbon levels compared with generalist or ruderal species; conversely, AMF species that invest preferentially in hyphal biomass and recalcitrant exudates over labile carbon compounds favor carbon sequestration, albeit potentially at the expense of mutualistic benefits to the host. (4) Assemblages of AMF taxa with complementary life-history traits-combining strong mutualists, carbon-investing species, and ruderal types are hypothesized to synergistically enhance both plant productivity and soil carbon sequestration, thereby reconciling the apparent trade-offs identified in the first hypothesis.

## 2 Research advances in AMF mediated soil carbon transformation under intercropping systems

AMF both form mutualistic associations with plant roots and enhance nutrient uptake, thereby improving crop growth efficiency. They also participate in soil organic matter decomposition and the carbon cycle, thus influencing soil carbon transformation. Studies have indicated that AMF affect soil carbon transformation through physical and chemical pathways. For instance, upon exposure to AMF, plant roots release certain substances, and AMF secrete compounds as well, establishing a bidirectional interaction (Basiru and Hijri, 2024). Once the association is established, AMF hyphae can promote soil carbon sequestration by physically entangling with and adhering to soil particles (Adhikari et al., 2023). Moreover, AMF secrete various enzymes that degrade soil organic matter, converting complex organic carbon into forms that plants can readily take up (Siddiqui and Pichtel, 2008). AMF can also promote the growth of microorganisms that fix atmospheric nitrogen. For example, AMF may induce changes in the expression of genes involved in nodule symbiosis, thereby increasing nodule formation in legumes (Gorgia and Tsikou, 2026). Long term field experiments and laboratory simulations worldwide have explored how AMF affect soil organic carbon decomposition, mineralization, and humification (Wei et al., 2019). Research on AMF has also received wide attention, especially regarding soil health management in agroecosystems. Researchers have found that AMF improve the efficiency of nitrogen and phosphorus uptake by crops, thereby reducing the need for chemical fertilizers and increasing soil organic matter accumulation, which is crucial for promoting sustainable agricultural development (Zhuang et al., 2025).

Long term positioning experiments have revealed that AMF community composition shift in response to intercropping, with ruderal species increasing under high disturbance regimes and competitive guilds dominating under low disturbance conditions (Zhang et al., 2022). Isotope tracing studies using ^13^CO_2_ pulse labeling have confirmed rapid transfer of photosynthetic carbon from plants to AMF hyphae in intercropped systems, with subsequent partitioning into hyphal biomass and soil residues (Johnson et al., 2002a; Kaiser et al., 2015). Moreover, approaches such as functional omics have identified AMF specific genes involved in carbon metabolism and exudate production, providing molecular level insights into species specific differences in carbon investment strategies (Basiru and Hijri, 2024).

## 3 Effects of intercropping systems on rhizosphere nutrients and carbon components

Evidence indicates that intercropping can improve soil physicochemical properties, maintain fertility and nutrient balance, and promote macroaggregate formation and organic matter retention on clay minerals, thereby enhancing soil carbon stability compared with monoculture (Liu et al., 2019). In many legume/grass intercropping systems, the effects on rhizosphere nutrients are driven by different crop combinations: legumes supply atmospheric nitrogen through fixation, which maximizes nutrient use and boosts agroecosystem productivity.

In intercropping systems, exudates such as amino acids and organic acids are released from diverse roots and residues, which increase microbial biomass supply, promote soil microbial community diversity, and ultimately accelerate soil carbon and nitrogen cycling (Lai et al., 2022). Studies also have shown that in intercropping systems, different crops can significantly increase the soil contents of nitrogen, phosphorus, potassium, and other nutrients by sharing soil resources and interacting with each other, while also altering soil organic matter content, thereby improving soil structure and fertility (Bai et al., 2022; Zhou et al., 2023; Xu et al., 2021). In addition, intercropping systems improve soil activity and carbon cycling, as well as increase microbial abundance and key enzyme activities. These changes collectively promote soil carbon sequestration, balance elemental fluxes, and enhance the supply of nutrients and trace elements (Ilakiya et al., 2023; Zhao et al., 2023).

Collectively, these findings indicate that intercropping systems can foster soil carbon sequestration and nutrient supply, largely through modulating rhizosphere microbial communities and enzyme activities, thereby promoting overall soil fertility and elemental cycling. Intercropping systems affect rhizosphere soil nutrients by improving nutrient cycling, increasing nutrient availability, and enhancing microbial activity. These effects help improve soil fertility, promote plant growth and development, and support soil health and ecological balance.

Intercropping influences soil carbon composition through multiple pathways. First, it alters microbial community structure and diversity, which in turn affects carbon transformation processes (Zhou et al., 2025). At the global scale, the soil organic carbon pool-estimated at 1,500-2,344 Gt, far exceeding atmospheric and vegetation carbon stocks (Batjes, 2014; Eswaran et al., 1993; Stockmann et al., 2013)-receives substantial inputs of plant photosynthetic carbon via AMF hyphal networks under intercropping (Averill et al., 2014; Frey, 2019; See et al., 2022; Hoosbeek et al., 2011). Among SOC fractions, active organic carbon is the most dynamic: highly mobile, chemically labile, readily oxidized and degraded by microorganisms, yet bioavailable and sensitive to changes in nutrient supply (Luo et al., 2020). Intercropping promotes SOC accumulation not only by improving soil quality and photosynthetic efficiency (Wang et al., 2025) but also through microbial processing of plant-derived organic matter (Picone et al., 2025). Notably, microbial residue carbon-particularly from fungi constitutes the dominant fraction of stable SOC, far exceeding living microbial biomass (Wardle, 1992; Liang et al., 2019). Under intercropping, root-driven microbial life-history strategies enhance both SOC levels and necromass quality, with crop diversity positively correlating with carbon sequestration. Despite these positive indications, the overall effects of intercropping on soil carbon composition remain context-dependent and require further investigation.

## 4 Effects of AMF on soil carbon transformation under intercropping systems

Arbuscular mycorrhizal fungi associate with over 70% of land plants, forming mutualistic symbioses in which plants supply photosynthetic carbon mainly as hexoses and fatty acids while fungi enhance host nutrient and water acquisition (Brundrett and Tedersoo, 2018; Khaliq et al., 2022; Johnson et al., 2002a; Kaiser et al., 2015). In intercropping systems, this partnership is particularly consequential for soil carbon dynamics.

AMF function as a biological conduit, channeling a substantial fraction of plant photosynthetic carbon into the soil via extraradical mycelium. This carbon is partitioned into four pools: living hyphae, hyphal residues, hyphal secretions, and CO_2_ respired by the fungus. A portion is allocated to hyphal biomass, another is lost through respiration, some is released as labile exudates, and the rest is sequestered in the SOC pool as stabilized residues (Johnson et al., 2002b; Grimoldi et al., 2006; Moyano et al., 2007). Carbon transported by AMF hyphae away from root zones reduces respiratory losses and enhances sequestration, a process most pronounced under nitrogen or phosphorus limited conditions, where fungal mediation of nutrient acquisition is most critical (Zhu and Miller, 2003; Treseder, 2004; Corrêa et al., 2015).

A key distinction is the differential effect of AMF on labile vs. recalcitrant carbon pools. Labile carbon, including simple sugars, organic acids, and amino acids released as hyphal exudates, is rapidly metabolized by soil microorganisms, inducing positive priming effects that accelerate native SOC decomposition. Recalcitrant carbon, including glomalin-related soil proteins (GRSP), chitinous hyphal walls, and melanized residues, exhibits prolonged persistence in soil, contributing to long term carbon stabilization (Basiru and Hijri, 2024). Quantitatively, GRSP accounts for 3-5% of SOC, reaching up to 40% in some ecosystems (Agnihotri et al., 2022), while fungal necromass contributes 24-40% of microbial residue carbon (Liang et al., 2019). Microbial residue carbon as a whole constitutes 33-62% of SOC, far exceeding the contribution of living microbial biomass (1-3%) (Wardle, 1992; Liang et al., 2019).

A growing body of evidence, however, highlights trade-offs between AMF-induced plant growth and SOC storage. Strong mutualists that mobilize mineral-associated organic matter to boost plant nutrition may accelerate SOC turnover, leading to net carbon loss. In contrast, species that invest in recalcitrant compounds, such as glomalin-related soil proteins and hyphal biomass favor long term carbon stabilization, though potentially at the cost of reduced mutualistic benefits to the host (Basiru and Hijri, 2024). However, this trade-off is not universal. Several studies have reported simultaneous increases in plant biomass and SOC under AMF inoculation, particularly in nutrient-limited soils where AMF enhancement of plant productivity leads to greater carbon inputs that offset decomposition losses (Treseder, 2004; Wang et al., 2025). These divergent outcomes are linked to fungal life-history strategies: ruderal species with fast growing, short lived hyphae contribute rapidly to necromass-derived SOC, while competitive guilds with extensive, long-lived hyphae promote soil aggregation and mineral-associated organic matter formation over extended timescales (Chagnon et al., 2013). Thus, AMF community composition not just presence or abundance, critically shapes soil carbon outcomes under intercropping.

The priming effect of AMF on SOC decomposition is a key mechanism that requires clarification. AMF can induce positive priming by releasing labile exudates that stimulate microbial growth and activity, accelerating the decomposition of native SOC. This effect is pronounced in nutrient-rich soils where microbial communities are energy-limited. Conversely, AMF can suppress priming in nitrogen-limited soils, where competition with saprotrophs for nitrogen restricts decomposer activity, or under conditions where recalcitrant carbon inputs (e.g., glomalin, hyphal residues) dominate and physically protect existing SOC through aggregate formation (Basiru and Hijri, 2024; Frey, 2019). The net priming outcome thus depends on soil nutrient status, AMF species identity, and the quality of carbon inputs.

**Figure 2:**
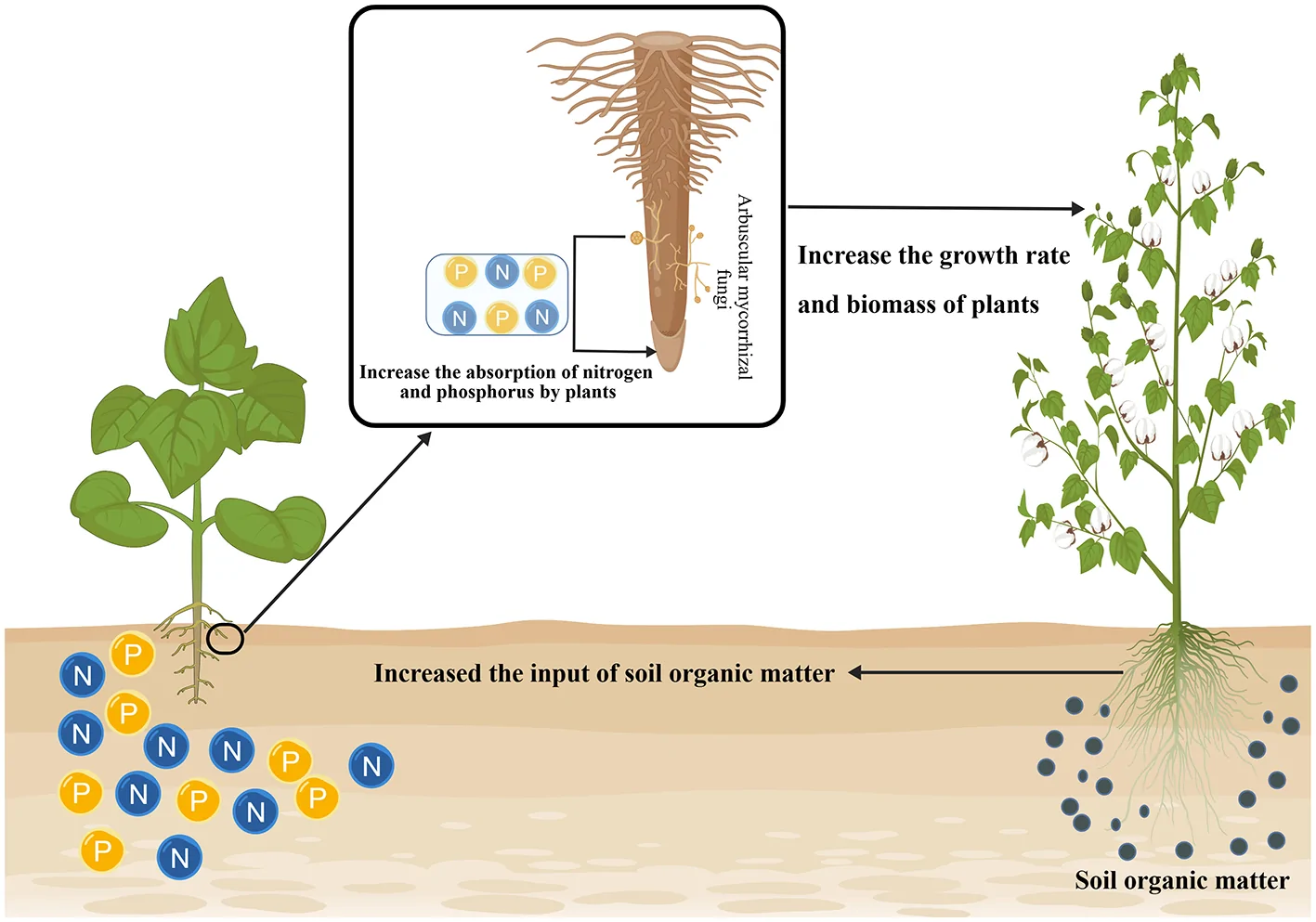
Conceptual diagram of AMF-mediated soil carbon transformation pathways in intercropping systems.

Beyond carbon transfer, AMF regulate soil carbon transformation through three complementary mechanisms (Figure 2). First, they enhance plant nutrient uptake (especially N and P), increasing biomass and rhizodeposition, which in turn fuels soil organic matter inputs and provides additional carbon substrates. Moreover, they modulate microbial community composition and activity, either stimulating or suppressing decomposer populations, thereby influencing the rate of organic matter mineralization (Yang et al., 2011). AMF hyphae physically enmesh soil particles, improve aggregation, and alter water vapor dynamics, all of which contribute to aggregate stability and physical protection of organic carbon (Ning et al., 2025). AMF hyphal networks also facilitate carbon transfer between intercropped plants, as demonstrated in watermelon/rice systems where AMF colonization increased interspecific carbon flow (Ren et al., 2013).

The net effect of AMF on SOC formation and persistence is modulated by a suite of abiotic and biotic factors. Environmental factors play a crucial role in shaping the balance between SOC stabilization and carbon loss. Under nitrogen or phosphorus limitation, AMF enhance nutrient acquisition and carbon allocation belowground, often resulting in increased SOC sequestration (Treseder, 2004; Corrêa et al., 2015). Drought stress can reduce AMF hyphal biomass and exudation, potentially limiting carbon transfer to soil; however, AMF may also improve plant drought tolerance, maintaining photosynthetic carbon supply under water-limited conditions (Sugiura et al., 2020). Elevated CO_2_ generally increases plant photosynthetic rates and carbon allocation to AMF, promoting greater hyphal biomass and residue inputs to SOC pools (Hoosbeek et al., 2011; See et al., 2022). Climate, soil nutrient status, and mineral composition interact with plant identity and AMF species to determine mycelial biomass, residue chemistry, exudate composition, and aggregate stability. Glomalin, a glycoprotein characterized by exceptional recalcitrance and secreted by arbuscular mycorrhizal fungi, constitutes a major fraction of active SOC, enhances aggregate stability, accumulates over prolonged timescales, and is closely tied to soil restoration potential (Wilson et al., 2009). Fungal residues, as a key component of microbial necromass, represent a considerable share of soil organic carbon-markedly outweighing the contribution of active microbial biomass, and are considered a principal conduit for long-term carbon stabilization (Wardle, 1992; Liang et al., 2019). Arbuscular mycorrhizal fungi reinforce this necromass reservoir via two complementary routes: directly, through mycelial turnover, and indirectly, by enhancing the formation of residual organic matter and its association with soil minerals.

Despite its recognized importance, direct empirical evidence that explicitly links AMF life-history traits to discrete carbon transformation pathways remains scarce; current inferences rely heavily on theoretical models and microcosm-based observations (Frey, 2019; See et al., 2022; Marschner et al., 2008). This gap calls for rigorous field-scale validation across diverse intercropping systems, varying crop combinations, and broad environmental gradients. Collectively, the available evidence indicates that AMF influence soil carbon dynamics in intercropping contexts through hyphal-driven carbon transfer, microbial community regulation, and physical stabilization of soil structure. Yet, the ultimate effect-both in magnitude and direction-is strongly contingent on the identity and composition of the AMF community, as well as on abiotic site conditions, which collectively underscore the critical need for system-specific research to underpin sustainable land-use decisions.

## 5 Significance of AMF-mediated soil carbon transformation for intercropping

In intercropping systems, where multiple plant species coexist in proximity, the regulation of carbon flow between plants and their associated microorganisms becomes particularly complex. AMF play a central role in this context, as they form hyphal networks that simultaneously connect multiple host plants, creating a shared carbon-exchange platform that is largely absent in monocultures. Within this framework, host plants exert molecular control over carbon allocation to AMF through the expression of genes such as acid invertases and hexosyltransferases, which modulate carbohydrate supply to the fungal symbiont and thereby influence soil CO_2_ efflux. This plant-mediated regulation is especially consequential in intercropping, where differential carbon investment among coexisting species can shape competitive dynamics and resource partitioning.

Beyond molecular regulation, AMF hyphae physically stabilize soil aggregates, promoting the structural protection of organic carbon-a mechanism that is particularly beneficial in intercropping systems, where root diversity and rhizodeposition are elevated. At the same time, AMF can accelerate the decomposition of labile organic matter by utilizing simple carbon compounds, thereby exerting a priming effect on soil carbon pools. This dual capacity-both stabilizing and destabilizing soil carbon-positions AMF as key modulators of carbon balance in intercropping soils. Consequently, AMF not only mediate carbon transfer and storage but also influence nutrient availability and interspecific interactions among intercropped plants, ultimately affecting system productivity, soil health, and resilience to environmental stress. By regulating both the physical protection and biological turnover of soil carbon, AMF serve as a critical biological nexus linking plant diversity, soil functioning, and ecosystem sustainability in intercropping systems.

## 6 Conclusions

Arbuscular mycorrhizal fungi function as a central biological hub in soil carbon dynamics within intercropping systems. They channel a substantial portion of plant photosynthetic carbon into the soil via extraradical hyphae, where it is partitioned among living biomass, residues, labile exudates, and respired CO_2_, a process amplified by the enhanced root diversity and hyphal network development characteristic of intercropping.

A key insight is the inherent trade-off between AMF-induced plant growth and soil organic carbon storage. Strong mutualists that mobilize mineral-associated organic matter to boost plant nutrition may accelerate SOC turnover, whereas species investing preferentially in recalcitrant compounds-such as glomalin-related soil proteins and hyphal biomass-favor long-term carbon stabilization, albeit often at the expense of mutualistic benefits. These divergent outcomes are closely linked to fungal life-history strategies: ruderal species contribute rapidly to necromass-derived SOC, while competitive guilds promote soil aggregation and mineral-associated organic matter formation over extended timescales. Consequently, AMF community composition, not merely presence or abundance, critically determines net carbon outcomes under intercropping.

Beyond carbon transfer, AMF regulate SOC through three complementary mechanisms: enhancing plant nutrient uptake (particularly nitrogen and phosphorus), modulating soil microbial community activity, and physically stabilizing soil aggregates. The net effect is modulated by a suite of abiotic factors-climate, soil nutrients, and mineral composition-and biotic factors such as plant identity and AMF species. Glomalin and fungal necromass emerge as major contributors to persistent SOC pools.

Despite these advances, direct empirical evidence explicitly linking AMF life-history traits to discrete carbon transformation pathways remains scarce; current understanding relies heavily on theoretical models and microcosm experiments. Rigorous field-scale validation is urgently needed across diverse intercropping systems and environmental gradients. Priority research directions include stable isotope tracing to quantify AMF contributions to SOC turnover, high-throughput functional profiling of AMF communities, and integration of these processes into predictive carbon models.

Based on the synthesized evidence, we propose some strategies to harness AMF for sustainable agriculture, including developing multi species AMF inoculants that combine mutualistic, carbon investing, and ruderal traits tailored to specific intercropping systems, optimizing legume/cereal row ratios, spacing, and planting schedules to enhance AMF colonization and hyphal network development, and integrating intercropping with reduced tillage and organic amendments to minimize soil disturbance while supplying carbon substrates for AMF growth. These approaches collectively reconcile productivity and carbon sequestration goals.

AMF play an indispensable role in intercropping by regulating both the physical protection and biological turnover of soil carbon. Their ultimate effect-whether enhancing sequestration or accelerating loss-depends on species identity, community composition, and environmental context. System-specific investigations are essential to guide sustainable agricultural practices that harness AMF for both productivity and climate change mitigation. Addressing these knowledge gaps will strengthen mechanistic understanding and support the design of resilient, carbon-smart intercropping systems.
